# Intuitive Cognition-Based Method for Generating Speech Using Hand Gestures

**DOI:** 10.3390/s21165291

**Published:** 2021-08-05

**Authors:** Eldad Holdengreber, Roi Yozevitch, Vitali Khavkin

**Affiliations:** 1 Department of Mechanical Engineering and Mechatronics, Ariel University, Ariel 40700, Israel; vitalyk@ariel.ac.il; 2 Navigation and Accessibility Research Center of Ariel University (NARCA), Ariel 40700, Israel; 3 Department of Computer Science, Holon Institute of Technology, Holon 58102, Israel; roiyo@hit.ac.il

**Keywords:** hand gestures recognition, muteness, speech disability, depth camera, Leap Motion Controller, speech interface, cognitive sensing, information entropy

## Abstract

Muteness at its various levels is a common disability. Most of the technological solutions to the problem creates vocal speech through the transition from mute languages to vocal acoustic sounds. We present a new approach for creating speech: a technology that does not require prior knowledge of sign language. This technology is based on the most basic level of speech according to the phonetic division into vowels and consonants. The speech itself is expected to be expressed through sensing of the hand movements, as the movements are divided into three rotations: yaw, pitch, and roll. The proposed algorithm converts these rotations through programming to vowels and consonants. For the hand movement sensing, we used a depth camera and standard speakers in order to produce the sounds. The combination of the programmed depth camera and the speakers, together with the cognitive activity of the brain, is integrated into a unique speech interface. Using this interface, the user can develop speech through an intuitive cognitive process in accordance with the ongoing brain activity, similar to the natural use of the vocal cords. Based on the performance of the presented speech interface prototype, it is substantiated that the proposed device could be a solution for those suffering from speech disabilities.

## 1. Introduction

Muteness at its various levels is a common disability [1]. This disability can be caused due to a number of factors such as nervous system diseases, stroke, sclerosis, autism, anatomical resections, and many more. There are a number of non-surgical supportive and alternative communication technology solutions, such as eye control communication (“Hawking language”), typing, handwriting, sign language using aids, etc. In most cases, these solutions lack the flexibility to become a decent substitute for human language and in other cases require prolonged learning of a new language (e.g., sign language). Simple handwriting can be a good solution, but one that does not allow verbal communication and is less suitable in cases where visual communication is irrelevant.

In recent years, a number of applications and interfaces have been developed to help people with speech disabilities. Many of the leading methods in the field are based on hand gesture recognition. Some studies have demonstrated the identification of general physiological gestures to vocal speech [2,3], and other studies showed a direct simultaneous translation from sign language to vocal speech [4,5,6,7]. In addition to sign language interpretation, hand gesture recognition [8] is attracting a growing interest due to its applications in many different fields, such as human–computer interaction, robotics, computer gaming, and so on. 3D hand pose estimation can also be achieved via neural networks [9,10]. The Leap Motion Controller device (*LMC*), first introduced in 2012, has opened new opportunities for gesture recognition [11]. The *LMC* is essentially a depth camera device that captures the movements of the hands with high accuracy [12,13]. Its companion algorithm enables the device (according to the manufacturer) to reach an accuracy level of ≈0.01 mm. Previous studies have also suggested the *LMC* can be a useful tool for translating sign language and hand gestures into sounds [14,15,16].

In this study, we propose a different approach for helping those suffering from speech disabilities. The proposed technology is based on the *LMC* for hands’ gesture recognition. Hand gestures are translated into vowels or consonants and those, in turn, are translated into syllables, according to the phonetic division (see Section 3 for a detailed explanation). This method relies on an intuitive approach to the vocal languages as words being comprised of syllables. The gesture-phonetic mapping goes coarsely like this: Hand gestures are divided according to different attitude (yaw, pitch, and roll) and position. Each attitude–position combination is uniquely linked to a vowel–consonant pair. A dedicated software plays this syllable in real time using speakers. There are several speech synthesis approaches and this paper restricts itself to concatenative synthesis approach [17].

Through a process of self-learning the user can intuitively communicate his/her desired “speech sounds” through the system, thus creating a working replacement for his/her dysfunctional speech system. The process itself works as follows: The user is taught the gesture–syllable map (which gesture is responsible for which syllable). Once the user gets the gist of it, we start the self-learning process. In this process, the user tries to create sounds and words using hand gestures and instant vocal feed-back from the system.

## 2. The Intuitive Cognition Speech Interface

In this section, we elaborate on the basic framework of the Intuitive Cognition Speech Interface (ICSP). The ICSP is comprised both from Hardware (the LMC and speakers) and software. The fusion between the two creates the speech interface.

### 2.1. Hardware

**Figure 1:**
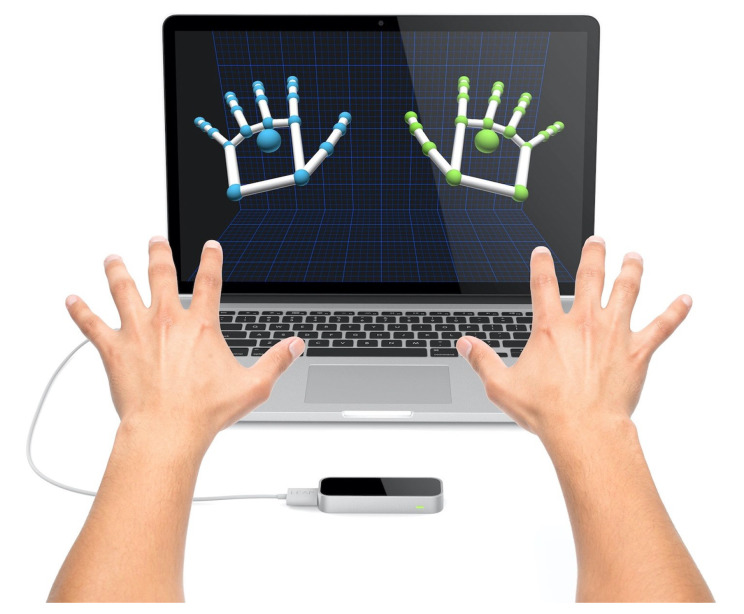
The Leap Motion controller (LMC) device showing a 3-D representation of both hands.

The *LMC* is a new consumer-grade sensor developed by Leap Motion (Leap Motion, http://www.ultraleap.com, accessed on 14 March 2021). It is primarily designed for hand gesture and finger position detection in interactive software applications (mainly gaming and 3-D CAD). Because of the current patent pending, only insufficient information on the underlying software’s geometrical or mathematical frameworks is available. Figure 1 shows a schematic view of the controller’s hardware setup. Differently from other devices (such as the Microsoft Kinect [18]), the *LMC* is explicitly targeted to hand gesture recognition and directly computes with high accuracy the hands’ positions and postures. The following figure shows both the controller itself and the 3D output of two hands simultaneously.

**Figure 2:**
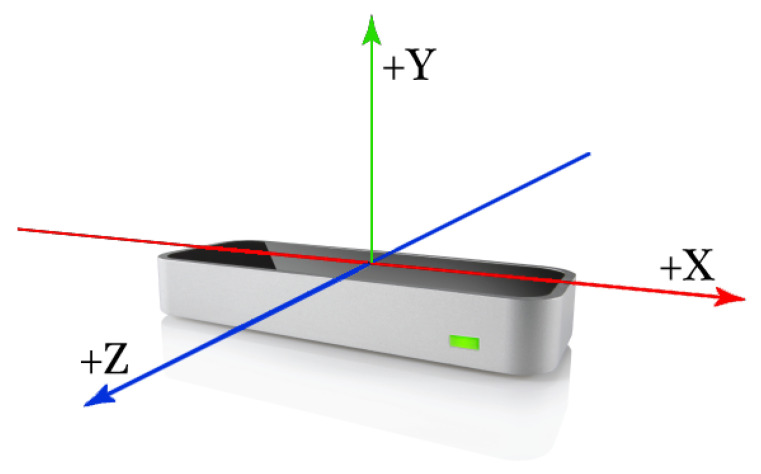
The LMC X-Y-X orientation.

The controller itself reports the position, velocity, and orientation vector of each finger. As depicted in Figure 2, the controller can detect the relative position of two (or more) hands with high precision and in 3D. The position of each hand’s palm is reported relative to the sensor’s center (0,0,0).

**Figure 3:**
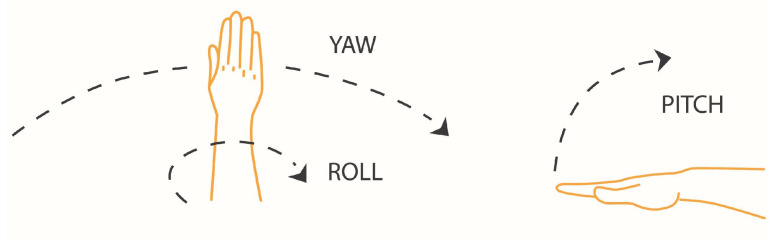
Hand’s different attitudes.

When the right hand moves away to the right, its x value increases. When the left hand moves away to the left, its x value decreases. The Y-axis is associated with the hand’s height relative to the sensor (there is no negative height). The Z-axis is associated with depth. For example, in Figure 1, when the hand approaches the screen, the Z value decreases. The LMC also provides data for hands’ yaw, pitch and roll. Figure 3 describes the difference between those attitudes.

In addition, the LMC can provide 2 more valuable pieces of information:

1. Hand Grip; it is an indication of how closed the fist is.
2. The thumb direction compared to the hand.

### 2.2. Software

The software was developed using Unity3D, a sophisticated game engine that allows for a relatively simple interaction with the *LMC*. An important feature of Unity is the ability to see in real time a 3D representation of the hands (as can be seen in Figure 1). Once the exact hands’ posture is captured, the software translates it to vowels and consonant, fuses them to a distinguished sound (syllable), and plays this sound via the speakers.

## 3. Sound Segmentation

Words are constructed from syllables, which, in turn, are constructed from vowels and consonants. This division is relevant for non-tonal languages such as (almost) all European languages, Hebrew, Arabic etc. [19]. Therefore, the basic building blocks are those two-vowels and consonants. Let us start with vowels.

### 3.1. Vowels

American English has seven vowel letters (A, E, I, O, U, Y, W), but those seven letters do not encapsulate the richness of all vowel sounds. For example, the letter **a** can be sound as /**a**/ (like in the word “f**a**ther”) or as /**æ**/ (like in the word “**a**pple”).

Those small but important distinctions were ignored in the proposed simulator. In the final section, we suggest some methods to tackle this challenge.

Vowels are produced by different right-hand postures.

### 3.2. Consonants

The American English language contains 21 different consonants letters which produce 24 consonants sounds in most English accents [20]. Because of the history of the English language, there is no neat one-to-one relationship between letter and sound. Some vowels can be used also as consonants. For example, in the word *yellow*, y is a consonant, but in the word *happy*, y is a vowel.

Consonants are expressed using the left hand’s posture. The combination of Left-Right hand creates syllables. The latter can be further divided to simple and advance syllables.

### 3.3. Simple Syllables

**Figure 4:**
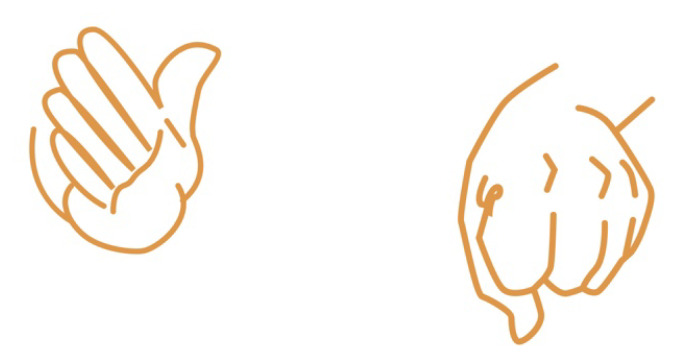
Hands posture for a “bee” sound.

We define simple syllables as syllables which contains a single vowel–consonant combination. For example, the sound “B-ee” (as in the word “*be*”) and the sound “A-r” (as in the word “*are*”) are simple syllables. They are simple as their sound can be produced from a simple right-left hand posture combination. Figure 4 demonstrates it well. The right hand posture for the sound “*ee*” is depicted in the left side, and the left-hand posture for “*B*” is depicted in the right side image. The sound “B-ee” is simply constructed from those two postures.

**Figure 5:**
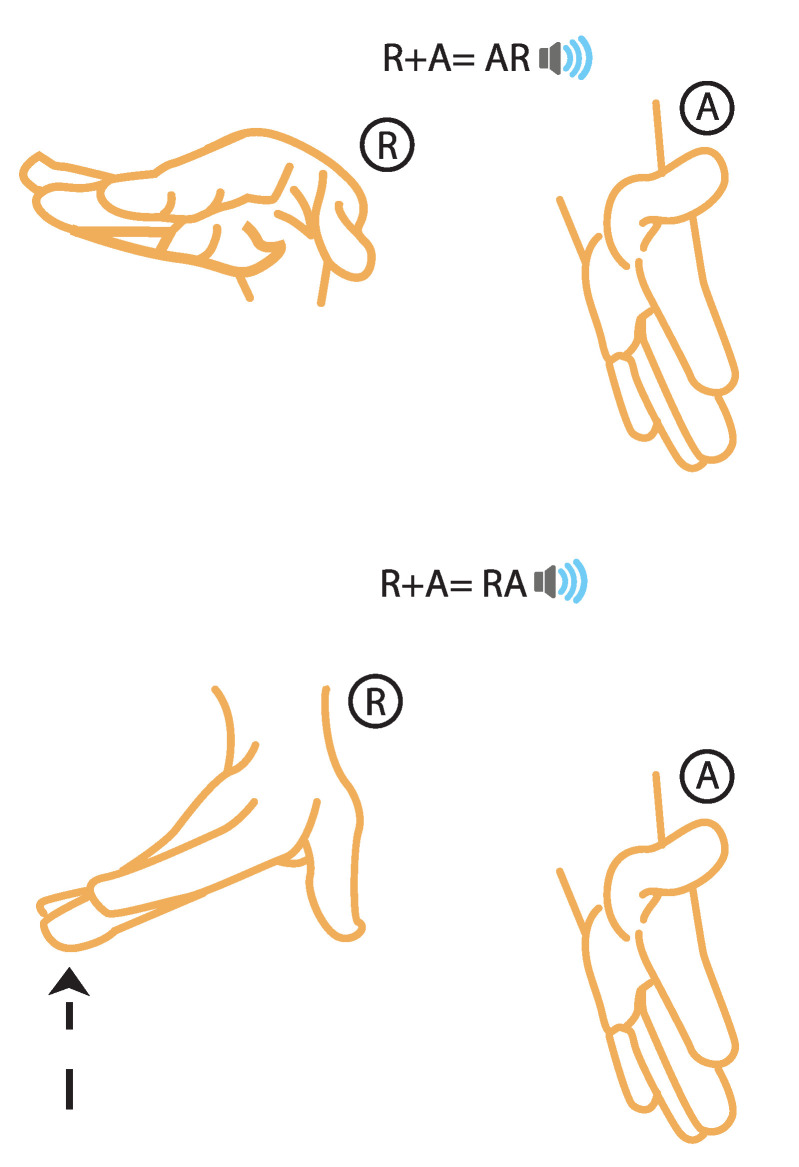
Hands’ posture for *AR* and *RA* sounds.

Although simple, those syllables do contain a slight complexity: the letter order. The combination R-A can be heard as “a-r” where the consonant follows the vowel (as in the word “are”) or as “r-a” where the vowel follows the consonant (as in the word “e*ra*”). This distinction is solved by the vertical position (y-axis) of the right hand. Thus, the syllable “A-r” will be expressed almost identical to the syllable “R-a” with the right-hand vertical position change. Figure 5 shows this difference.

### 3.4. Advanced Syllables

Many words cannot be broken down to a simple syllables sequence. The words Dad, Mom, Want, Father, and Dog to name only a few, are more complex. Their complexity lies in the additional consonant (usually at the end). For example, the word “dog” is comprised of a single (complex) syllable with two consonants (“d” and “g”) wrapping a vowel (“o”).

These words can be expressed using the proposed system as a combination of a simple syllable and a single consonant. For example, the word “dog” can be comprised of the simple syllable “do” and the consonant ”g”. Although the proposed solution is not ideal (the word dog is pronounced differently than “do-g”), the word can be easily understood. Another important feature this method enables is the small numbers of hand postures to memorize. Moreover, given some knowledge about the distribution and entropy of the English language, one can improve the system even more.

## 4. Entropy

Information entropy is a profound concept in the field of communication. The name was coined by Claude Shannon in a pioneering paper [21]. In a nutshell, the entropy of a system can be computed as

(1) $$H\left(x\right)=-\Sigma_{i=1}^{N}P\left(x_{i}\right)log_{2}P\left(x_{i}\right)$$

where *N* is the number of different symbols, and $p(x_{i})$ is the probability (or frequency) of i-th symbol. One can think of this equation as the average number of bits necessary to communicate a message. This message can represent an arbitrary distribution. When the distribution is uniform, information entropy gets its maximal value.

What is the entropy of the English alphabet? Assuming 27 letters (26 letters plus a “space” character) and a uniform distribution among all letters, the entropy is ≈4.75. It means that on average it would take 4.75 bits to communicate a single letter (for 32 uniform symbols, the entropy H(x)=5 bits as $2^{5}=32$). This is also called the “zero-order” model of English. However, the English alphabet is not distributed uniformly. For example, the probability of the letter “e” is 100 bigger than the probability of the letter ‘z’. In fact, Shannon himself found that when taking those different probabilities into account, the entropy decreases to H(x)=4.219.

The above figure (4.219 bits) is also called the “first-order” model of English. The assumption of independence (zero memory) is also incorrect. Some letters follow other letters frequently; others not at all (e.g., “u” must follow “q”). One can compute the likelihood of digrams (two-letter combinations), trigrams, etc. Adding digrams to the computation gives a second-order model; adding trigrams gives a third-order model. Table 1 shows the most frequent digrams and trigrams in the English language [22].

A “third-order model” yields 2.77 bits per symbol. The actual entropy is the “limit” of this process of taking higher and higher order models. The entropy figure is important because this paper strives to construct an alternative speech system. A key aspect in such system is to ease the pronunciation of frequent sounds (“ent”). Those frequent sound are found using entropy.

Some of the trigrams in the table are merely sounds (“ive”, “ent”) but some of them are also words (“and”, “for”, “our”). It is important to bear in mind that third-order sequences were calculated for written English, thus, the pronunciation (or sound) of these trigrams can be completely different, based on the context. For example, the trigram ION sounds different in the word “on**ion**” and in the word “celebrat**ion**” and the trigram IVE sounds different in the word “f**ive**” and in the word “effect**ive**”.

## 5. Experiments and Results

In order to properly communicate words and phrases using the system, one must practice it thoroughly. However, this demand can be relaxed with a moderate escalation. Another important thing to bear in mind is that this paper aims solely to present a proof of concept. Past research confirms that alternative forms of communications (e.g., switching the entire visual field upside down) can become, with practice, intuitive. In order to evaluate the performance of the proposed device, a comparison was made through signal processing speech analysis between synthetic words created by the device and spoken words [23,24,25].

### 5.1. Phase 1

Phase 1 is devoted to simple words. Such words are fabricated only from unique combinations of a single vowel and a single consonant.

#### 5.1.1. The Word “Banana”

**Figure 6:**
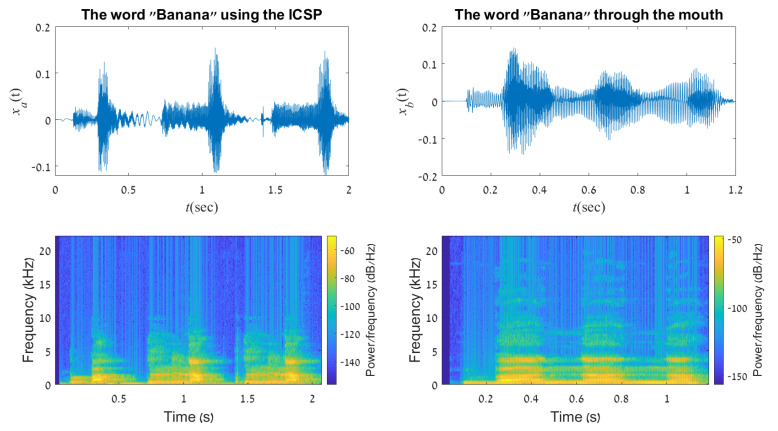
A representation of the word “Banana” created using the ICSP (**left graph**) and the human mouth (**right graph**). Top graphs represents the audio signals in the time domain. Down graphs are the frequency spectrum of the audio signals as it varies with time.

For example, the word “Banana” can be divided into the following syllabus sounds: “Ba-Na-Na”, as explained in Section 3.3. The participant is informed regrading which hands posture produces which sound and then he/she is instructed to convey simple words using the system. The conveyed word is then recorded and compared vs a normal pronunciation of the word. The left upper graph in Figure 6 shows the representation of the word “Banana” as a function of time as created using our framework. The right upper graph, demonstrates the same word as created using the human mouth. One can see that the duration of the ICSP word (2[sec]) is relatively long compared to that spoken by the mouth (1.2[sec]) [26]. The smoothing between the three syllables demonstrates the continuity of the spoken word compared to the three separated parts of the signal. Sound can also be represented as a frequency spectrum of an audio signal as it varies with time, this is called a spectrogram [27,28]. A spectrogram of sound is created from a time signal using the Fast Fourier Transform (FFT). The lower two images in the figure represent the word’s spectrogram. Dark blue corresponds to low amplitudes and the brighter colors up through orange correspond to progressively stronger or louder amplitudes. Each sound is divided into short time frames of 20 ms (2000-point windows) with a 512-point overlap between successive frames. Implementing Fourier transforms on these consecutive frames can help us obtain a good approximation of frequencies across the time domain. A Hamming window is applied to each frame to significantly reduce the spectral leakage before conducting FFT. The squared-difference mean between the two signals is very low (≈0.0001), which indicates high correlation, thus, the meaning of the ICSP word can be grasped easily.

#### 5.1.2. The Word “Daddy”

**Figure 7:**
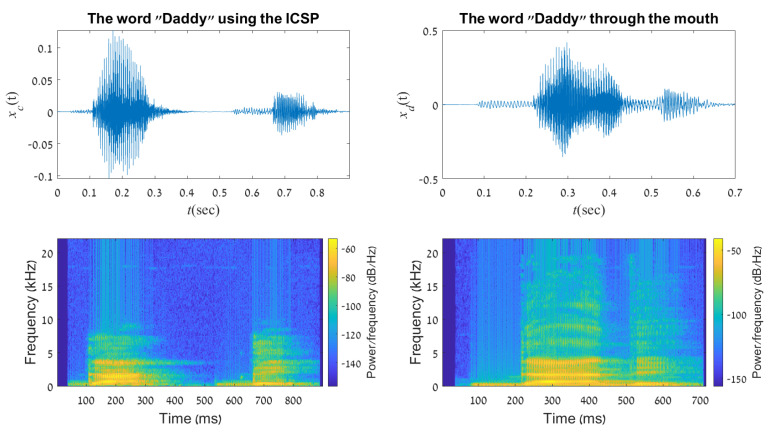
A representation of the word “Daddy” in time domain and frequency spectrogram, created using the ICSP (**left column**) and the human mouth (**right column**).

A sound output of the word “Daddy” can be depicted in Figure 7. The lower two images in the figure represent the word’s frequency domain. Again, the figure shows similar results to Figure 6. As in the previous word, the obtained squared-difference mean is low (≈0.003). However, this figure demonstrates roughly the same duration for both words. The signal spectrogram graphs show that the two produced words contain mainly frequencies of up to 4 kHz, so that 99.9% of the signal energy is concentrated in this frequency range. The high correlation between the two demonstrates the preservation of the spectral signature of the produced word. Note that the words generated through the ICSP depends on the user level of skill, as the ability to speak through the device is an acquired skill over time. In addition, in order to get better continuity in the words spoken through the ICSP, a real-time adaptive filter can be applied for use. Another important thing to remember is that the spectral comparison serves only as a mathematical aid. The foremost goal is to create speech that can be understood by lay persons, thus, a statistical evaluation is also offered later on in Section 5.3.

### 5.2. Phase 2

In the second phase we introduce more complex words. Again, first the participant in informed regarding the correct posture for each sound and then instructed to convey words.

#### 5.2.1. The Word “Thing”

**Figure 8:**
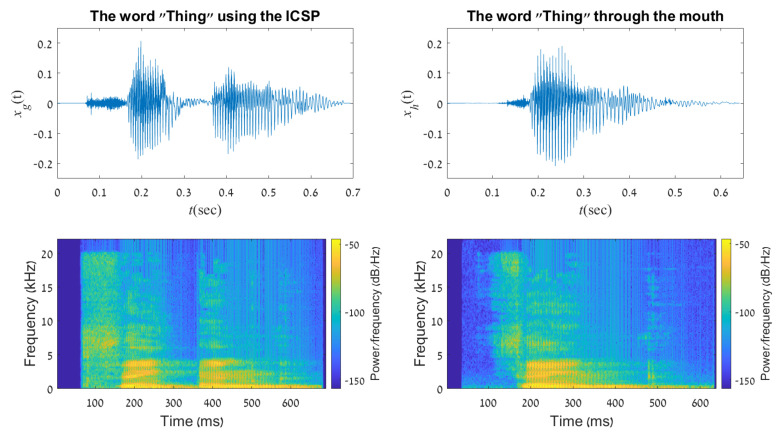
A representation of the word “Thing” in time domain and frequency spectrogram, created using the ICSP (**left column**) and the human mouth (**right column**).

As was shown before (Section 4), although the trigram “ING” is not a simple syllable, it is quite common in the English language, thus, a dedicated posture was uniquely assigned to it. The word “thing” is comprised from the sounds “thi” and “ing” in conjunction. Again, one can see the inevitable break between the two sounds on the upper left in Figure 8. The duration is ≈0.1 s and 99.5% of the signal energy is concentrated between frequency ranges up to 5 kHz.

#### 5.2.2. The Word “Event”

**Figure 9:**
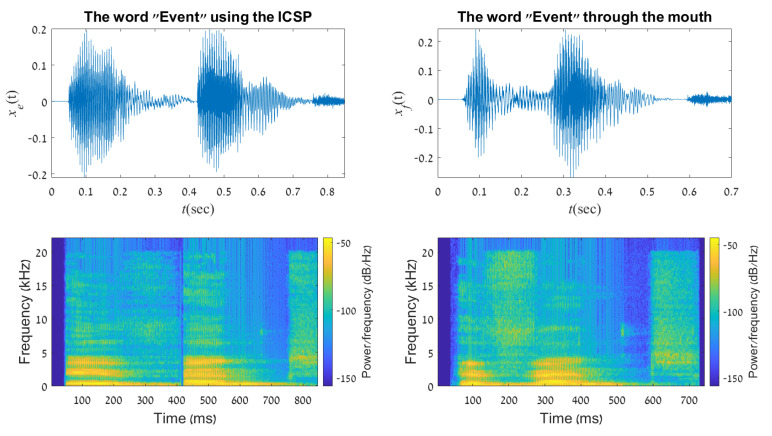
A representation of the word ”Event” in time domain and frequency spectrogram, created using the ICSP (**left column**) and the human mouth (**right column**).

The word “event”, as opposed to the word “thing”, is a two-syllable word (e-vent); thus, the break between the two syllables is quite similar on both words (top left and top right in Figure 9). One can find that the spectral components of those two words are with a very high match. It is indeed reflected in the ability to recognize those two words with a high degree of accuracy, as shown in the next section.

### 5.3. Statistic Analysis

A mandatory part of any communication protocol is the receiver’s ability to understand the transmitted message. Up until this point we have focused on how to efficiently generate words using the ICSP. In this subsection, we focus on the other end of the rope: are these synthetically generated words and phrases understandable? We have generated eight different words and a one single sentence, played them to 45 mature subjects, all in the age range of 20 to 40, and checked what did the subjects hear. The subjects’ native language is not English but Hebrew. The words are listed below.


Want                Thing                Pizza                

Happy               Event               Banana               

Carrot               Apple               You are Happy               


Table 2 shows the comprehension rate of each word/phrase. The first column represent the generated word/phrase. The second column represent the accuracy rate, i.e., the percentage of subjects who understood the correct word. For example, 53% of the subjects understood the word “want” (first word in the column). It means that 24 out of 45 subjects got this word. The two right columns represent the second best most common word and the overall accuracy of the two most common guesses. In other words, if one did not get the correct phrase, what was the second most probable phrase? In the above example, this word is “won’t” which is extremely similar to the correct word. 68% of the subjects heard the word “want” or “won’t”. The average accuracy (first and second guesses) score for the entire set is ≈78.5%.

Although the overall accuracy rate is not perfect for some of the words (“thing” (72%), “carrot” (59%)), they can be understood from the context. Conclusions cannot be deduced from a single example but we tend to believe that the high accuracy rate for the last phrase (“you are happy”) is also connected to this very point. This phenomenon is similar to automatic transcript algorithms (speech to text) that correct words backwards according to their context (just from the context, one can easily distinguish “want” from “won’t”).

## 6. Conclusions and Future Work

An intuitive cognition-based approach for playing vocal sounds through hands gestures recognition, using depth camera detection, is presented in this paper. The proposed device is capable of creating suitable vowels and consonants, consistently with a predetermined hand rotation movements. For smooth playback of the sounds and for a convenient user interface, the vocal entropy distribution and efficient software algorithmic were implemented. The prototype performances, served as a proof-of-concept, indicates that the proposed device has high potential as a solution for people with speech disabilities, while future research might show this method can bypass the traditional speech regions in the human brain. Future work might includes the aid of machine learning methods for classification and filtering [29,30].

## Figures and Tables

**Table 1:** Most frequent digrams and trigrams in the English language.

Digrams	Trigrams
EN	ENT
RE	ION
ER	AND
NT	ING
TH	IVE
ON	TIO
IN	FOR
TR	OUR
AN	THI
OR	ONE

**Table 2:** Comprehension accuracy rate table.

Comprehension Accuracy Rate			
**Word/Phrase**	**Acc [%]**	**2nd Best Guess**	**Overall Acc [%]**
Want	53	Won’t	68
Thing	24	Thinking	72
Pizza	71	Pisa	75
Happy	100	-	100
Event	82	You and	86
Banana	88	-	88
Carrot	48	Carrots	59
Apple	62	Appeal	66
You are happy	93	-	93

## Data Availability

The data presented in this study are available on request from the corresponding author.
